# Psychological distress in single fathers and mothers – a Swedish population-based study

**DOI:** 10.1177/14034948251332507

**Published:** 2025-04-10

**Authors:** Maria Unenge Hallerbäck, Anu Molarius, Linn Karlsson, Karin Sonnby

**Affiliations:** 1 Centre for Clinical Research, Region Värmland, Karlstad, Sweden; 2 Faculty of Medicine and Health, Örebro University, Örebro, Sweden; 3 Department of Public Health Sciences, Karlstad University, Karlstad, Sweden; 4 Centre for Clinical Research, Region Västmanland, Västerås, Sweden

**Keywords:** Single parents, mental health, parental support, population studies, Sweden

## Abstract

**Aims:**

The primary aim of the present study was to explore the prevalence of psychological distress among single fathers and single mothers in comparison to parents living together, and the factors contributing to the differences between single and partnered parents. A secondary aim was to investigate the perceived need for parental support in relation to severe psychological distress in these groups.

**Methods:**

A survey questionnaire was sent to a random population sample in Sweden in 2022 and 5750 parents aged 18–69 years participated. The outcome was severe psychological distress, measured by the Kessler-6 (scores ⩾13). Associations between single parenthood and severe psychological distress were analysed with multiple logistic regression, adjusting for age group, economic difficulties, social support, risk consumption of alcohol and need for parental support.

**Results:**

Severe psychological distress was more common among single fathers (age-adjusted odds ratio (OR): 2.2; 95% confidence interval (CI): 1.4–3.5) and mothers (age-adjusted OR: 2.4; 95% CI: 1.8–3.3) than among partnered fathers and mothers. The main explanatory factors for the difference were economic difficulties and lack of social support, accounting together for 75% of the excess of severe psychological distress in single fathers and 64% in single mothers. Risk consumption of alcohol among both single and partnered fathers was also associated with severe psychological distress. Being in need of more parental support, for example, from maternity/child health care or family centres was associated with severe psychological distress among all parents, regardless of partnership status and gender.

**Conclusions:**

**Single parents had a higher prevalence of severe psychological distress than partnered parents, mainly explained by economic difficulties and the lack of social support. Both among single and partnered parents, the need for more parental support was associated with severe psychological distress.**

## Background

Globally, the number of single parents is increasing, and Sweden is one of the countries with the highest percentage of single parent families [1]. In the OECD (Organisation for Economic Co-operation and Development) countries, nearly 15% of all children live with one parent, ranging from 5% in Greece to 26% in the United States [2], and almost 10% of all children live in reconstituted households [2]. In Sweden, about 25% of all parents are not living with the other parent, and 35% of the children of parents not living together, live equally much with both parents [1].

Even though most single parents are single mothers, a societal shift, indicating an increasing number of fathers taking on the role of sole caregiver for children, has also been observed [3]. The proportions of single fathers (of all fathers) in previous population-based studies were 3% in South Korea, 4% in Germany, 5% in Canada and New Zealand and 7% in Sweden, whereas the proportion of single mothers varied from 6% in South Korea, 14% in Sweden, 21% in Germany to 24% in New Zealand [1,4-7]. In the United States, the number of single fathers increased by more than 50% between 1990 and 2020 [8].

Previous studies have shown that impaired mental health, including higher prevalences of depressive symptoms, suicidal ideation, anxiety, and substance use, is more common among single parents than among partnered parents [4, 9-12]. In Sweden, an association between child living arrangements and the mental health of parents has been reported [1]. Several factors have been found to contribute to mental health in single parents. Both economic difficulties [13] and a lack of social support [14] are associated with impaired mental health and have been linked to the impaired mental health in single parents in comparison to partnered parents [1, 4-7, 15]. Economic difficulties may be a consequence of underlying societal conditions hampering opportunities to combine professional work with parental responsibilities [1, 6].

Moreover, child welfare systems tend to focus on maternal caretakers and their needs, and often disregard the contributions from the fathers [16 ]. A meta-synthesis of qualitive studies on parenting experiences showed that single fathers felt that social assistance systems favoured women and that single mothers were offered more credibility, resources and opportunities. The fears, needs and struggles of single fathers tended not to be considered [17].

Further, an association between impaired parental mental health and decreased mental health in children has been shown, stressing the need for adequate parental support limiting the negative consequences for vulnerable groups of parents like single parents, and indirectly, their children [18].

Previous studies have used different measures of impaired mental health and controlled for different socioeconomic, social and lifestyle factors [4, 9-12]. In Sweden, Fritzell et al. [1] reported that single mothers had a higher prevalence of symptoms of worry and anxiety than partnered mothers, whereas this was not the case for single fathers. However, their study used data from the Swedish Survey of Living Conditions 2008–2013 and had a partly different aim. Moreover, to the best of our knowledge there are no previous studies about the association between mental health and perceived need of parental support among single and partnered parents.

The aim of the present study was to explore the prevalence of psychological distress among single fathers and mothers in comparison to partnered fathers and mothers in Sweden, and the factors contributing to the differences between single and partnered parents. A secondary aim was to investigate the perceived need for parental support in relation to psychological distress.

## Methods

**Figure 1.**
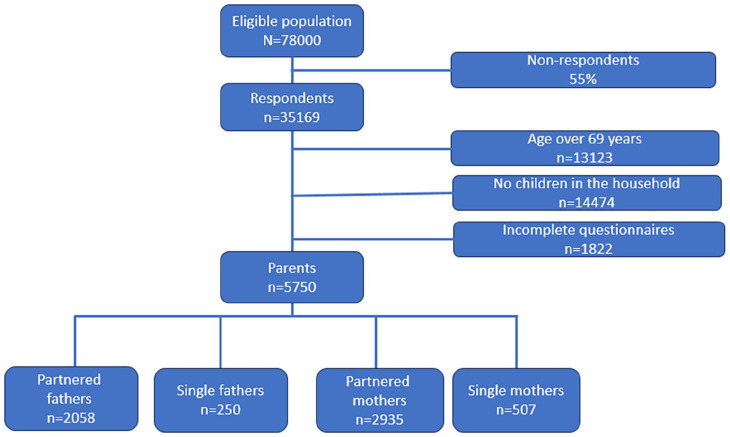
Flowchart of the survey sample and the study population.

The study is based on a survey questionnaire (Life and Health study) [19] sent to a random population sample of 78,000 individuals aged 18 years or over in five counties (Sörmland, Uppsala, Värmland, Västmanland and Örebro County) in Sweden (see Figure 1). The survey was carried out in February–May 2022. The aim of the survey was to monitor living conditions, lifestyle factors and health in the general population. The sampling frame was the population register at Statistics Sweden, the statistical administrative authority in Sweden, covering all inhabitants of the study area. Data collection was discontinued after two postal reminders. The overall response rate was 45%. The area investigated included 55 municipalities with over 1 million inhabitants in the central part of Sweden. The current study is based on a subsample constituted of all parents aged 18–69 years answering the questionnaire (*n* = 5750).

The outcome in the current study was psychological distress, measured by the Kessler-6, a widely used and validated screening tool for non-specific psychological distress [20, 21]. It includes six questions about how often the respondent during the past 30 days felt . . . 1) nervous, 2) hopeless, 3) restless or fidgety, 4) so depressed that nothing could cheer one up, 5) that everything was an effort, and 6) worthless. The response options were All the time/Most of the time/Some of the time/A little of the time/None of the time, with the scores ranging from 4 to 0, respectively. The total score thus ranged from 0 to 24. A cutoff of ⩾13 points is used to define people with severe psychological distress (i.e. those likely to have a diagnosable mental illness and associated functional limitations) [20, 21].

Family status was based on the question: ‘With whom do you share a home, that is, who do you live with during most of the week?’. The response options were ‘Nobody’, ‘Parents/siblings’, ‘Spouse/partner’, ‘Children younger than 18 years’, ‘Children 18 years or older’, and ‘Other adult’ (more than one answer could be given). Those who answered ‘Children younger than 18 years’ were considered to be parents and included in the current study. If the respondent also gave the answer ‘Spouse/partner’ he/she was categorised as a partnered parent, otherwise the respondent was categorised as a single parent. Of 757 single parents, 35 persons were living with parents/siblings and 37 persons with other adults.

Since economic difficulties, social support and alcohol consumption are related to impaired mental health [4, 13, 14, 22] we estimated their possible contribution to differences in severe psychological distress between single and partnered parents. Economic difficulties and social support were measured with questions identical to those in the national public health survey of the Public Health Agency of Sweden [21]. The question about economic difficulties was ‘During the last 12 months, have you ever had difficulty in managing the regular expenses for food, rent, bills etc.?’ (‘No’, ‘Yes, once’, ‘Yes, more than once’). The alternatives were dichotomised into ‘No’ and ‘Yes’. Social support was measured with the question, ‘Do you have anyone you can share your innermost feelings with and confide in?’ (Yes/No).

Alcohol consumption was measured using Alcohol Use Disorders Identification Test-C (AUDIT-C): a widely used and validated screening instrument of alcohol use [23]. It comprises three questions on the frequency of consumption (Never to 4+ times per week), the amount consumed on a typical day when drinking (1–10 units) and how often an individual consumed 6 or more units if female or 8 or more units if male on a single occasion in the last 12 months. The response options for the first and the third questions gave 4, 3, 2, 1, or 0 points, and the options to the second question gave 0, 1, 2, 3, or 4 points. The test can thus give a maximum of 12 points. The following cutoffs for risk-drinkers were used: 6–12 points for men and 5–12 points for women [24].

Need for parental support was measured with the question: ‘Do you need support in your role as a parent (e.g. support from maternity/child health care, family centres, student health care, associations, or other actors)?’. The response options were ‘Yes, and I get the support I need’, ‘Yes, but I could use more support’, and ‘No’.

The data on gender and age are based on registry data from Statistics Sweden. After adding the registry data, the material was pseudonymised before it was sent to the regions for further processing. Authorisation from the Swedish Ethical Review Authority was obtained (Dnr 2021-05814-01). The data is protected according to the Public and Privacy Act (2009: 400, Chapter 24, Section 8).

Differences in the prevalence of severe psychological distress between single and partnered parents were tested using chi-squared test. Univariate odds ratios (ORs) for severe psychological distress were calculated for single parents in comparison to partnered parents. The analyses were run separately for fathers and mothers. To assess the contribution of related factors we carried out logistic regression analyses in different steps. In the second step, age group was added into the model. In the third step, economic difficulties and social support were added into the model to estimate whether these factors could explain the difference observed in the age-adjusted model. In the fourth step, alcohol consumption and, in the last step, need for parental support was added into the model. The results are reported as ORs and 95% confidence intervals (95% CIs). The proportion of missing data varied between 0 and 1.6%. All analyses were carried out using SPSS, version 28.0.

We assessed the contribution of economic difficulties and lack of social support on the excess odds of severe psychological distress of single parents by explained fractions (%) [4]. Explained fractions were calculated as ((OR2–1) –(OR3–1))/(OR2–1), where OR2 is the OR of the single parent from the logistic regression model adjusting for age group and OR3 is the OR from the logistic regression model adjusting for age group, economic difficulties, and social support. The contributions of alcohol consumption and need for parental support were not assessed since adjusting for them did not change the ORs for single parents.

## Results

A total of 5750 parents participated, of those 40% were fathers (see Figure 1). Among the 757 single parents, there were 250 (33%) fathers. The proportion of single fathers was 11% among all fathers and the proportion of single mothers was 15% among all mothers.

Table I displays the background characteristics of single and partnered parents. A larger proportion of single parents reported economic difficulties and lack of social support compared to partnered parents. Single parents had a larger proportion with risk consumption of alcohol compared to partnered parents. Furthermore, there was a larger proportion of single mothers compared to partnered mothers who reported that they needed more parental support than they had access to.

**Table I.** Background characteristics of the study population.

	Total		Fathers			Mothers		
Variables	*N*		Single parent	Partnered parent		Single parent	Partnered parent	
*N*	5750		250 (4.3%)	2058 (35.8%)		507 (8.8%)	2935 (51.0%)	
		%	%	%	*p*-value*	%	%	*p*-value*
Age group					<0.001			0.101
18–29 years	617	10.7	8.8	7.1		9.3	13.7	
30–49 years	4170	72.5	63.2	69.8		73.2	75.1	
50–69 years	963	16.7	28.0	23.1		17.6	11.2	
Economic difficulties					<0.001			<0.001
No	4822	84.0	74.7	87.3		68.2	85.2	
Yes	917	16.0	25.3	12.7		31.8	14.8	
Social support					<0.001			<0.001
Yes	5161	89.9	76.8	87.3		81.0	92.6	
No	583	10.1	23.2	12.7		19.0	7.4	
Alcohol consumption					0.054			0.004
Not risk-drinker	5157	91.1	84.1	88.4		90.3	93.8	
Risk-drinker	583	8.9	15.9	11.6		9.7	6.2	
Need for parental support					0.309			0.006
No	4485	78.8	80.1	82.3		71.9	77.5	
Yes, and I get the support that I need	741	13.3	10.8	11.2		15.6	14.1	
Yes, but I need more support	463	8.1	9.1	6.5		12.6	8.5	

* *p*-value for difference between single and partnered parents.

Severe psychological distress (measured by the Kessler-6 non-specific distress scale) was more common among single parents compared to partnered parents among both genders (*p* < 0.001) (Table II). The prevalence of severe psychological distress was slightly higher among single mothers compared to single fathers, but this difference was not statistically significant (*p* = 0.213).

**Table II.** Prevalence (%) of severe psychological distress among single and partnered parents.

Variables	Total		Fathers			Mothers		
	*N*	%	Single parent	Partnered parent	*p*-value*	Single parent	Partnered parent	*p*-value*
Severe psychological distress					<0.001			<0.001
Yes	392	6.8	10.9	5.2		14.1	6.2	
No	5332	93.2	89.1	94.8		85.9	93.6	

* *p*-value for difference between single and partnered parents.

Next, logistic regression analyses were performed separately for mothers and fathers, exploring the explanatory variables’ association with severe psychological distress (Table III). The OR for severe psychological distress for single parents was over 2 among both genders. The analyses showed that younger parents reported higher proportions of severe psychological distress compared to older parents. In addition, economic difficulties and lack of social support were strongly associated with severe psychological distress. Economic difficulties and lack of social support explained a large proportion of the difference between single and partnered parents in severe psychological distress (see the difference between the analytic steps in OR2 (adjusted for age group) and OR3 (adjusted for age group, economic difficulties, and social support) in Table III). The proportion of the excess OR between single and partnered parents explained by economic difficulties and lack of social support (explained fraction) was 75% among fathers and 64% among mothers. Among fathers, though not among mothers, risk consumption of alcohol was associated with severe psychological distress. Moreover, fathers and mothers who reported a need for more parental support showed increased odds of severe psychological distress. However, mothers who reported that they did not need parental support, had an OR below 1 for severe psychological distress compared to those who reported a need for parental support. Alcohol consumption and need for parental support did not however further explain the difference between single and partnered parents in severe psychological distress.

**Table III.** Odds ratios for severe psychological distress (95% confidence interval in parenthesis) by parent group and explanatory factors.

Variables	Fathers					Mothers				
	OR1	OR2	OR3	OR4	OR5	OR1	OR2	OR3	OR4	OR5
Parent group										
Partnered	1	1	1	1	1	1	1	1	1	1
Single parent	**2.1 (1.3, 3.4)**	**2.2 (1.4, 3.5)**	1.3 (0.8, 2.1)	1.2 (0.7, 2.1)	1.2 (0.7, 2.1)	**2.3 (1.7, 3.2)**	**2.4 (1.8, 3.3)**	**1.5 (1.1, 2.1)**	**1.5 (1.1, 2.1)**	**1.5 (1.1, 2.1)**
Age group										
18–29 years	-	1	1	1	1	-	1	1	1	1
30–49 years	-	**0.5 (0.3, 0.8)**	**0.5 (0.3, 0.8)**	**0.5 (0.3, 0.9)**	**0.5 (0.3, 0.9)**	-	0.8 (0.5, 1.1)	0.9 (0.6, 1.4)	0.9 (0.6, 1.4)	0.9 (0.6, 1.3)
50–69 years	-	**0.4 (0.2, 0.7)**	**0.4 (0.2, 0.8)**	**0.4 (0.2, 0.8)**	**0.4 (0.2, 0.8)**	-	**0.6 (0.3, 1.0)**	0.8 (0.4, 1.3)	0.7 (0.4, 1.3)	0.7 (0.4, 1.3)
Economic difficulties										
No	-	-	1	1	1	-	-	1	1	1
Yes	-	**-**	**4.0 (2.7, 6.0)**	**4.1 (2.7, 6.1)**	**3.8 (2.5, 5.8)**	-	-	**4.3 (3.3, 5.8)**	**4.3 (3.2, 5.7)**	**3.9 (2.9, 5.2)**
Social support										
Yes	-	-	1	1	1	-	-	1	1	1
No	-	-	**7.7 (5.2, 11.5)**	**7.9 (5.3, 11.8)**	**7.1 (4.7, 10.6)**	-	-	**3.4 (2.4, 4.7)**	**3.4 (2.4, 4.8)**	**3.3 (2.3, 4.6)**
Alcohol consumption										
Not risk-drinker	-	-	-	1	1	-	-	-	1	1
Risk-drinker	-	-	-	**1.9 (1.1, 3.1)**	**1.9 (1.1, 3.2)**	-	-	-	1.5 (0.9, 2.4)	1.6 (1.0, 2.6)
Need for parental support										
No	-	-	-	-	1.1 (0.6, 2.2)	-	-	-	-	**0.6 (0.4, 0.9)**
Yes, and I get the support I need	-	-	-	-	1	-	-	-	-	1
Yes, but I need more support	-	-	-	-	**3.3 (1.5, 7.3)**	-	-	-	-	**1.8 (1.1, 2.8)**

*Note*. OR1: crude odds ratio; OR2: odds ratio adjusted for age group; OR3: odds ratio adjusted for age group, economic difficulties, and social support; OR4: odds ratio adjusted for age group, economic difficulties, social support, and alcohol consumption; OR5: odds ratio adjusted for age group, economic difficulties, social support, alcohol consumption, and need for parental support. Statistically significant ORs are shown in bold type. Hypen denotes here in the tables are all not included in the model.

## Discussion

In the present study, severe psychological distress was more common among single fathers and mothers than among partnered fathers and mothers. The main explanatory factors for this difference were economic difficulties and lack of social support. Further, risk consumption of alcohol among both single and partnered fathers was associated with severe psychological distress. Perceived need for more parental support was associated with increased odds of severe psychological distress among all parents, regardless of partnership and gender.

Our results indicating a higher prevalence of severe psychological distress among single parents in both genders compared to partnered parents are in line with former population-based studies from Germany [6], New Zealand [5] and South Korea [4]. Further, studies from Belgium [15] and Canada [7] found impaired mental health among single parents compared to other household or family compositions.

In the present study, economic difficulties and lack of social support were more common among single than partnered parents and explained 75% and 64% of the excess OR of severe psychological distress among single fathers and mothers, respectively. This is in line with the results of previous studies from Europe [2, 6, 15], Canada [7], New Zealand [5] and South Korea [4] that found that socioeconomic factors and social support are the main explanatory factors for impaired mental health in single parents. Rattay et al. [6] in Germany, Gisle et al. [15] in Belgium and Kong et al. [4] in South Korea found socioeconomic factors and levels of social support to be the main explanatory factors for impaired mental health in single parents. However, these factors only partly explained the difference between single and partnered parents, which is in line with the results of the present study. A similar result for single mothers and fathers in Sweden was also found by Fritzel et al. [1].

In contrast to the present study, Chiu et al. [7], investigating mental health among single fathers in Canada, found socioeconomic factors (low income and unemployment) to be the only explanation for the impaired mental health compared to partnered fathers. Additionally, Fritzell et al. [1] found joint custody (also known as joint residency) to be a further explanatory factor for impaired mental health in single fathers, after adjustment for socioeconomic factors. Different measures of socioeconomic factors and mental health in the studies may have contributed to these dissimilarities. Moreover, the custody status of the parents was not measured in the present study.

In Belgium, Gisle et al. [15] found the increased odds of suicidal behaviour among single parents were partly explained by socioeconomic factors. However, suicidal behaviour is a different construct from severe psychological distress, therefore a direct comparison is hampered. The study by Chiu et al. [7] from Canada reports the mental health of single fathers compared to single mothers and partnered fathers based on one question on self-rated mental health, which may not measure severe psychological distress as measured with the Kessler-6 in the present study.

Further, the present study found risk consumption of alcohol among both single and partnered fathers to be associated with severe psychological distress. In South Korea another pattern was found by Kong et al. [4], namely that increased alcohol consumption among both single fathers and single mothers, but not among partnered parents, was associated with impaired mental health. Different instruments for the assessment of alcohol consumption, and possibly also cultural differences concerning alcohol consumption and the gender as well as the age of the study participants (18–69 years in our study and 30–59 years in Kong et al.’s research) may have contributed to these differences. The studies by Fritzel et al. [1], Rattay et al. [6] and Gisle et al. [15] did not report on the relationship between the mental health of single and partnered parents and alcohol consumption.

In the present study, perceived need for more parental support (e.g. from maternity/child health care, family centres, student health care, associations, or other actors) was associated with increased odds of severe psychological distress among all parents, regardless of partnership status and gender. No other study has explored the self-reported need for parental support in relation to measures of mental health among single and partnered parents. It is notable that the need for more parental support was associated with severe psychological distress in single mothers as well as in single fathers.

The results of the present study must be interpreted in the light of several limitations. Firstly, there was a large proportion of non-respondents. The response rate was lower among younger subjects, in men and among those with a low educational level than in other groups. This may have reduced the representativeness of these groups. Therefore, it is unknown whether the present study sample is representative of the whole population of parents in the study area. Most probably there are more parents with severe psychological distress among the non-respondents than among the respondents, therefore the results of the present study may be conservative [25].

Secondly, the definition of single parent allowed parents not living with a partner/spouse to share a household with their own parents/siblings or other adults, which may have influenced the results. However, the proportion of single parents living with own parents/siblings or other adults constituted a very small proportion among the single parents. In addition, the interpretation of the question about ‘living with children most of the time’, which was the basis for identification of single parents, may not be homogenous. Therefore, it is unknown how much time the single parents really lived with their children and whether there were any differences between single mothers and single fathers in the interpretation of the question. A Swedish demographic report about the residence of children with parents living apart found that twice as many fathers than mothers report that their children had alternating residence [26]. Possible explanations mentioned were that more parents living with their children most of the time had answered the questionnaire or that parents over- or underestimated the time they lived with their children.

Thirdly, there was a low number of single fathers, making the analyses more vulnerable to random variations. Therefore, as few explanatory factors as possible were included in the analysis models. However, the number of single fathers was higher in the present study than in several of the other previous studies [4, 5, 15]. Moreover, the data were based on self-reports, which may have introduced reporting bias. For example, a gender bias in reporting mental health symptoms among men compared to women has been observed (in addition to gender differences in the prevalence of impaired mental health, with higher prevalence among women than men) [27]. This may also have influenced the results of the present study. Furthermore, the measure for need of parental support was not part of a validated instrument with explored psychometric properties, even though the question showed face validity. Furthermore, the cross-sectional study design impeded the possibility of analysing any causes of severe psychological distress. It is not possible to know whether the severe psychological distress preceded or followed single parenthood. Regardless of the origin of psychological distress, it was associated with an increased need for parental support. Future studies should investigate longitudinal associations between single parenthood and mental health. Longitudinal studies are also important to explore the causal link between single parenthood, severe psychological distress and the health of children in these families.

In addition to the limitations, the study also had several strengths. Firstly, it was based on a survey sent to a random sample representing a large population, covering five counties with both larger cities and more rural areas in the central part of Sweden. Secondly, the data were relatively new, from 2022. Thirdly, the definition of single parent in the present study was similar to several previous studies [1, 4, 6, 7]. Finally, the study used Kessler-6 as a measure of severe psychological distress, which is a validated instrument with good psychometric properties for self-report in population-based studies [20]. Moreover, the Kessler-6 has been shown to quantify psychological distress through the use of different cutoffs in order to differentiate between moderate and severe psychological distress in relation to mental health care utilisation, impairment and substance use [28]. In addition, AUDIT-C is a validated instrument for risk consumption of alcohol [29].

## Conclusion

In summary, the present study, based on a large population survey in Sweden, supports the finding that single parents have a higher prevalence of severe psychological distress than partnered parents, mainly explained by economic difficulties and a lack of social support. Moreover, both among single and partnered parents, the perceived need for more parental support was associated with severe psychological distress. To the best of our knowledge, the present study is the only one investigating both severe psychological distress and the need for parental support among single and partnered parents. These findings may be of interest for planning health care policies for parents. Policies to increase the economic safety and social support among single parents and parental support among all parents may have the potential to improve the mental health of single and partnered parents.
